# Robotic-Assisted Total Gastrectomy for Gastric Cancer After Coronary Artery Bypass Grafting Using the Right Gastroepiploic Artery: A Case Report

**DOI:** 10.7759/cureus.67446

**Published:** 2024-08-21

**Authors:** Ippei Yamana, Takahisa Fujikawa, Yuichiro Kawamura, Suguru Hasegawa

**Affiliations:** 1 Surgery, Kokura Memorial Hospital, Kitakyushu, JPN; 2 Gastroenterological Surgery, Fukuoka University Hospital, Fukuoka, JPN

**Keywords:** robotic gastrectomy, gastroepiploic artery, coronary artery bypass grafting, total gastrectomy, gastric cancer surgery

## Abstract

The right gastroepiploic artery (RGEA) is frequently used in coronary artery bypass grafting (CABG) for right coronary artery bypass requiring long-term patency. We experienced a case of upper-third advanced gastric cancer after CABG using RGEA. The absence of enlarged lymph nodes (LNs) or distant metastasis was confirmed through computed tomography (CT), and the RGEA graft remained patent according to coronary CT angiography. Based on these findings, the patient underwent robotic total gastrectomy while preserving the RGEA graft without infra-pyloric LN dissection. We suggested that caution should be exercised to avoid injury to the graft during gastrectomy, and robotic surgery could contribute to safely preserving the RGEA. We should consider the decision to dissect the infra-pyloric LN for the patient's safety and curability.

## Introduction

The use of the right gastroepiploic artery (RGEA) as a graft for right coronary artery bypass was first reported in 1987 by Pym et al. [1]. Thereafter, RGEA was frequently used for severely stenosed right coronary arteries as a graft [2].

However, there is a problem when it comes to gastric cancer patients who have undergone coronary artery bypass grafting (CABG) using the RGEA. The problem is whether to perform infra-pyloric lymph node (LN) dissection or not. Preserving the RGEA is necessary, but performing the infra-pyloric LN dissection requires careful judgment and precise maneuvers to avoid injury to the RGEA.

Robotic surgery was introduced to overcome the limitations of conventional laparoscopic surgery and expand the use of minimally invasive surgery [3]. Concerning the robotic gastrectomy, it reduces morbidity compared to the laparoscopic approach [4]. Robotic surgery offers advantages such as articulated functionality, autonomous camera operation, and three-dimensional (3D) magnification. There are few case reports of robotic gastrectomy with preservation of an RGEA graft for gastric cancer patients with a history of RGEA graft use [5]. Here, we present a case of robotic-assisted total gastrectomy safely performed for gastric cancer after CABG using RGEA.

## Case presentation

**Figure 1:**
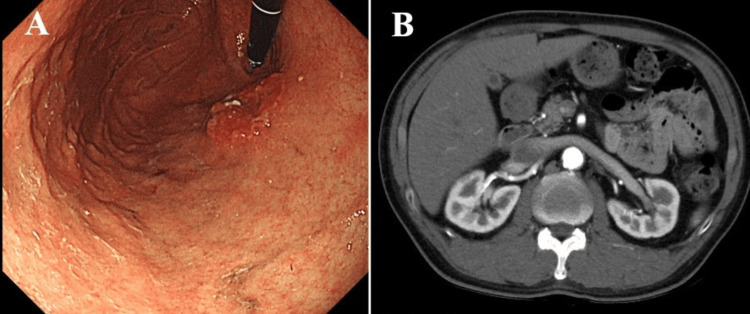
Gastrointestinal endoscopy and contrast-enhanced CT findings. (A) Gastrointestinal endoscopy revealed an advanced gastric tumor measuring 30 × 30 mm in the anterior wall of the cardia. (B) Contrast-enhanced CT showed no enlarged LN or distant metastasis. CT, computed tomography; LN; lymph node

**Figure 2:**
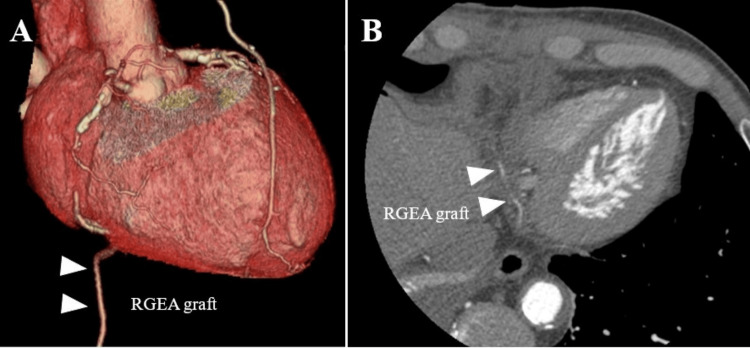
Coronary CT angiography findings before gastrectomy. (A, B) Coronary CT angiography showed that the RGEA graft remained well patent (arrows: RGEA graft). RGEA, right gastroepiploic artery

A 67-year-old male was referred to our hospital for the treatment of coronary artery disease. He underwent three-vessel CABG, involving a bypass between the right coronary artery and the RGEA, between the left anterior descending branch and the left internal thoracic artery, and between the intermediate branch and the right internal thoracic artery. After CABG, he received dual antiplatelet therapy. One month after CABG, he was admitted to our hospital because of hematemesis. Gastrointestinal endoscopy revealed a gastric tumor measuring 30 × 30 mm in the anterior wall of the cardia (Figure 1A). A biopsy confirmed a diagnosis of poorly differentiated adenocarcinoma. Computed tomography (CT) showed no enlarged LN or distant metastasis (Figure 1B). The clinical stage was classiﬁed as T2N0M0, stage I. Coronary CT angiography showed that the RGEA graft remained well patent (Figure 2A and Figure 2B).

**Figure 3:**
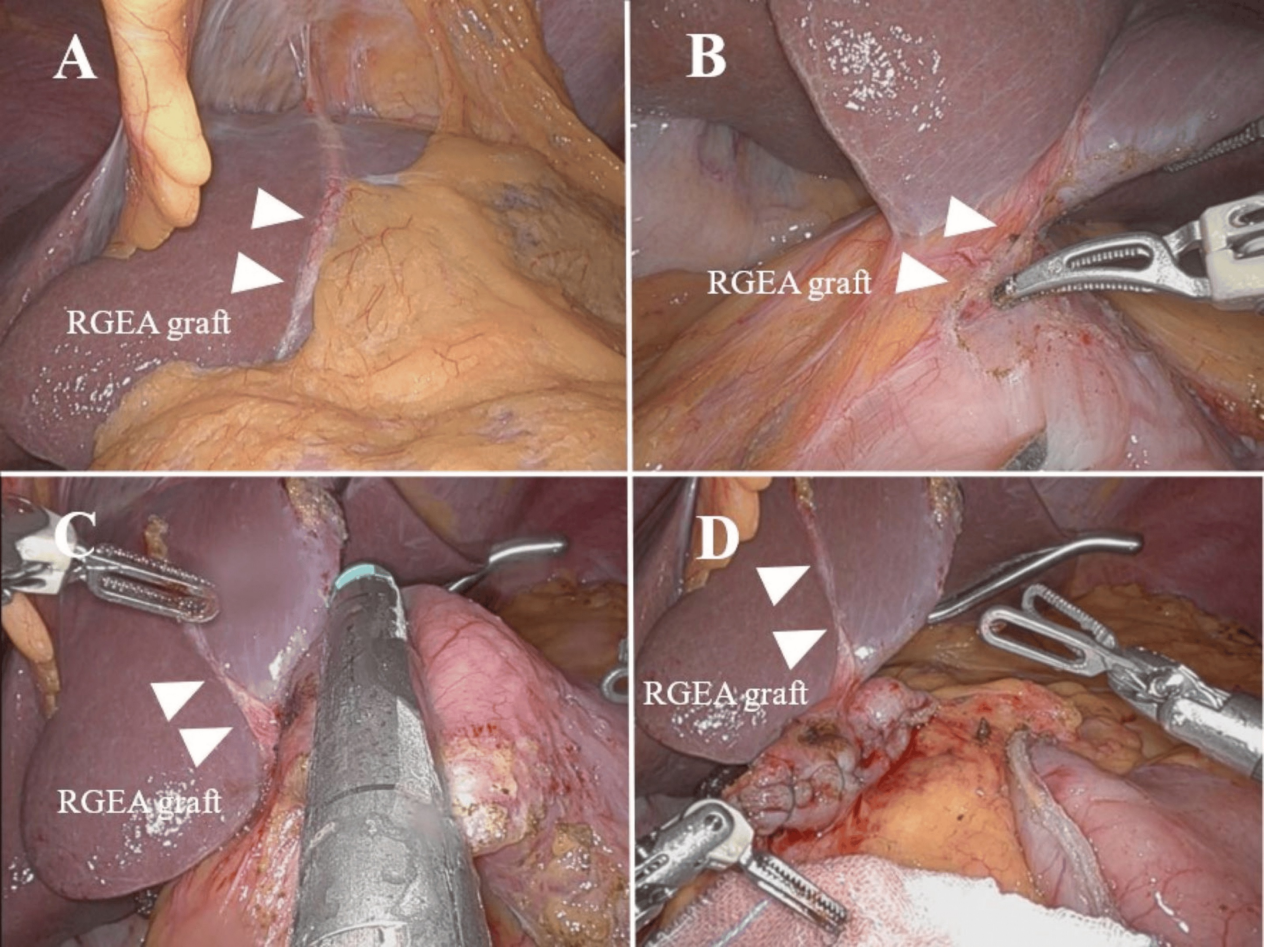
Operative findings. (A) The RGEA graft was easily recognized intraoperatively on the left lobe of the liver (arrows: RGEA graft). (B) The RGEA graft was passed over the pyloric ring (arrows: RGEA graft). (C) The stomach was cut off at the oral side of the pyloric ring while preserving the RGEA graft (arrows: RGEA graft). (D) The staple lines were reinforced with sutures (arrows: RGEA graft). RGEA, right gastroepiploic artery

Two months after CABG, a robotic-assisted total gastrectomy using the Da Vinci Xi surgical system (Intuitive Surgical, Inc., Sunnyvale, CA, USA) was performed. Cardiovascular surgeons were on standby during the gastrectomy in case of a graft injury. Intraoperatively, the location of the RGEA graft was easily confirmed on the anterior side of the gastric pyloric ring and the left lobe of the liver (Figure 3A and Figure 3B). The infra-pyloric LNs were not dissected to avoid graft damage. Because the RGEA graft passed over the pyloric ring, the stomach was cut off at the oral side of the pyloric ring while preserving the RGEA graft (Figure 3C). The staple lines were reinforced with sutures (Figure 3D). The procedure of robotic total gastrectomy preserving RGEA graft is shown in Video 1. Operation time and console time were 452 minutes and 395 minutes, respectively. The blood loss was 25 mL. The pathological diagnosis was poorly differentiated adenocarcinoma. The ﬁnal stage was classiﬁed as T2N1M0, IIA. No postoperative complications occurred, and he was discharged 14 days after the operation.

**Video 1:** The surgical technique around the RGEA graft. The RGEA graft was well patent. Infra-pyloric LN dissection was not performed because the probability of LN metastasis was low. The RGEA graft was successfully preserved. RGEA, right gastroepiploic artery; LN, lymph node

He did not receive adjuvant chemotherapy due to chronic renal dysfunction and chronic heart failure. Unfortunately, he developed multiple liver metastases six months after surgery, and he demanded the best supportive care. One year after surgery, he passed away due to the underlying disease.

## Discussion

The gastrectomy for gastric cancer patients with a previous history of CABG using RGEA requires careful attention because if the RGEA is injured, it can lead to significant coronary failure [6-13]. Therefore, it is necessary to carefully evaluate the balance between safety and curability. To date, the safety of robotic gastrectomy for preserving the RGEA graft has not been established. In our case, because the RGEA graft was well patent on preoperative coronary CT angiography, we decided to preserve the RGEA graft and not dissect the infra-pyloric LN. Consequently, we could successfully perform a robotic-assisted total gastrectomy preserving RGEA graft safely.

There are two strategies for gastrectomy in patients with a history of RGEA graft use. First, a gastrectomy is performed without dissection of the infra-pyloric LN if possible or with dissection of the infra-pyloric lymph LN while carefully preserving the RGEA graft [6-10]. Second, if it is not possible to keep the RGEA, coronary revascularization is done before surgery through redo CABG or percutaneous coronary intervention, or it is done during surgery through simultaneous CABG or more alternative grafting [11-13]. Concerning the necessity of infra-pyloric LN dissection for gastric cancer patient, the likelihood of infra-pyloric LN metastasis is reported to be low in the case of upper-third gastric cancer [14,15]. Ri et al. reported only one case of infra-pyloric LN metastasis out of 167 upper-third gastric cancer patients with cT2 lesions or cT3/T4 lesions [14]. Yura et al. reported no infra-pyloric LN metastasis out of 202 upper-third gastric cancer patients with cT2 or T3 lesions [15]. In our case, the patient did not undergo infra-pyloric LN dissection, and the RGEA graft was preserved. This decision was made because there were no enlarged infra-pyloric LNs observed on preoperative CT, and the likelihood of metastasis to the infra-pyloric LN was low due to the location of the tumor in the upper-third gastric region.

There are two techniques for harvesting the RGEA during CABG: the skeletonized method and the pedicled method [16]. In the case of the pedicled method, it is often difficult to identify RGEA because of adhesions to the surrounding fatty tissue, with a risk of RGEA injury. On the other hand, in the case of the skeletonized method, identification of RGEA is relatively easy. In our case, it was easy to identify the skeletonized RGEA graft on the pyloric ring and the left lobe of the liver.

There have been several case reports of open gastrectomy for gastric cancer patients after CABG using RGEA [6-13], whereas there is only one report of such patients undergoing robotic-assisted gastrectomy [5]. Minimally invasive surgery has advantages over open surgery [17]. In particular, we consider the advantages of robotic-assisted gastrectomy to be the 3D magnification effect and stable camera work. These advantages may prevent an inadvertent RGEA graft injury. These characteristics have enabled us to perform a robotic-assisted total gastrectomy for the gastric cancer patient who previously underwent CABG using RGEA safely.

## Conclusions

We successfully performed a robotic-assisted total gastrectomy for a gastric cancer patient who had previously undergone CABG using the RGEA. Robotic advantages, such as articulated functionality, autonomous camera operation, and the 3D magnification effect, could contribute to safely preserving RGEA during surgery. When deciding whether to perform infra-pyloric LN dissection or not, we should consider a balance between safety and curability for the patient based on information about the progression and location of gastric cancer and whether or not the RGEA graft is patent. We hope that our case report may help develop strategies for the gastric cancer patient who previously underwent CABG using RGEA.
